# Moderate drought causes dramatic floral transcriptomic reprogramming to ensure successful reproductive development in *Arabidopsis*

**DOI:** 10.1186/1471-2229-14-164

**Published:** 2014-06-13

**Authors:** Xuan Ma, Noor Liyana Sukiran, Hong Ma, Zhao Su

**Affiliations:** 1 Department of Biology and the Huck Institutes of the Life Sciences, the Pennsylvania State University, University Park, State College, PA 16802, USA; 2 Intercollege Graduate Program in Cell and Developmental Biology, the Pennsylvania State University, University Park, State College, PA 16802, USA; 3 State Key Laboratory of Genetic Engineering and Institute of Plant Biology, Institute of Genetics, Center for Evolutionary Biology, School of Life Sciences, Fudan University, Shanghai 200433, China; 4 Institutes of Biomedical Sciences, Fudan University, Shanghai 200032, China; 5 Current address: School of Biosciences and Biotechnology, Faculty of Science and Technology, Universiti Kebangsaan Malaysia, 43600 UKM Bangi, Selangor, Malaysia

**Keywords:** Moderate drought, Severe drought, Reproductive development, Transcriptome, Arabidopsis

## Abstract

**Background:**

Drought is a major constraint that leads to extensive losses to agricultural yield worldwide. The potential yield is largely determined during inflorescence development. However, to date, most investigations on plant response to drought have focused on vegetative development. This study describes the morphological changes of reproductive development and the comparison of transcriptomes under various drought conditions.

**Results:**

The plants grown were studied under two drought conditions: minimum for successful reproduction (45-50% soil water content, moderate drought, MD) and for survival (30-35%, severe drought, SD). MD plants can produce similar number of siliques on the main stem and similar number of seeds per silique comparing with well-water plants. The situation of SD plants was much worse than MD plants. The transcriptomes of inflorescences were further investigated at molecular level using microarrays. Our results showed more than four thousands genes with differential expression under severe drought and less than two thousand changed under moderate drought condition (with 2-fold change and q-value < 0.01). We found a group of genes with increased expression as the drought became more severe, suggesting putative adaptation to the dehydration. Interestingly, we also identified genes with alteration only under the moderate but not the severe drought condition, indicating the existence of distinct sets of genes responsive to different levels of water availability. Further *cis*-element analyses of the putative regulatory sequences provided more information about the underlying mechanisms for reproductive responses to drought, suggesting possible novel candidate genes that protect those developing flowers under drought stress.

**Conclusions:**

Different pathways may be activated in response to moderate and severe drought in reproductive tissues, potentially helping plant to maximize its yield and balance the resource consumption between vegetative and reproductive development under dehydration stresses.

## Background

The increasing world population (up to 7 billion in 2010) suggests a growing demand in crop production. Agricultural productivity is inevitably impacted by environmental stresses, such as drought, salinity, heat and cold [1]. Many of these abiotic factors can cause loss of yields partially resulting from dehydration of plant cells. Despite the abundance of water on earth, most of the water resources are not usable for irrigation due to salinity. Thus, an increasing number of investigations has focused on the mechanisms enabling plants adaptation to dehydration. Dehydration resistance consists of two main categories: dehydration avoidance or dehydration tolerance [2]. Dehydration avoidance is defined as the plant capacity to maintain cellular hydration in spite of stress and plants could achieve it by maintaining soil moisture, limiting water use (WU), and osmotic adjustment (OA). Dehydration tolerance is defined as the relative capacity to maintain normal function even in a (partially) dehydrated state, which is also viewed as a secondary defense against desiccation. This mechanism is not commonly observed other than in seed embryo and the only main exception occurs during certain stages of grain filling under drought [3].

Drought, the most direct reason leading to plant dehydration, has been studied for years. It could impact plants at molecular, cellular, physiological and biochemical levels and can severely affect multiple developmental process, including seed germination [4], seedling growth [5], root development [6] and later leaf development [7-9]. In many flowering plants, the emergence of flowers coincides with drought stress during summer. To ensure successful reproduction, flowering plant must possess mechanisms that protect flowers from severe dehydration. However, only a few studies have examined reproductive development under drought conditions at molecular level [10,11].

Another challenge for scientists studying drought is how to control water availability. It is known from both agricultural experience and experimental studies that varying degrees of water shortage could impact crop development and yield to different extents [12]. A few studies tried to calculate the minimum water requirement in certain regions and proposed to enhance the capacity in dealing with drought using water management [13,14]. However, field studies on drought potentially have substantial limitation for several reasons: 1) the difficulty of controlling soil water content accurately; 2) delay of drought effects on plant due to the variation of evaporation rate and soil content; and 3) substantial deviation due to variation of nutrients in soil. Therefore, it is difficult to estimate the minimum water requirement for plant survival or fertility from field studies.

On the other hand, studies in the lab could allow relatively accurate control of the water amounts to explore the mechanisms that plants employ to survive. By reducing the water supply, many genes have been found to be involved in complex drought responses, including both ABA-dependent and ABA-independent drought-responsive pathways [1,15,16]. Further studies revealed additional key components in these pathways, including transcription factors belonging to the bZIP, AP2/ERF, and MYB families [17-19]. With the help of transcriptomic profiling, more and more drought-responsive genes have been reported, especially in the model plants whose genomic information is available, such as *Arabidopsis*, rice and maize [1,20,21]. However, those studies often focused on vegetative tissues [20,22].

A recent study in our lab has shown the impacts of severe drought on reproductive development, such as reduced flower number and size, and fewer seeds [11]. In addition, detailed morphological analyses showed that the development of both male and female reproductive organs was affected by drought, resulting in ovule abortion, failure of flowers to open, abnormal anther development and delayed elongation of the filaments and stigmatic papillae cells. Further examination of the inflorescence transcriptomes under well-watered and severe drought conditions indicated that the floral transcriptome underwent dramatic reprogramming during severe drought treatment [11]. However, as only severe drought was applied in the previous study, it was not clear what are the effects of different extent of drought stresses on reproductive development and transcriptomes.

Here, to understand the impacts of different magnitude of drought stress on reproductive development, we treated the *Arabidopsis* plants with different drought severities soon after the bolting stage (around the time of the first opening flower) and observed their morphological changes. We further investigated the changes of inflorescence transcriptome under a moderate degree of drought. We collected inflorescences from treated plants at different times after moderate drought treatment and used the mRNA samples from the inflorescences for microarray experiments. The differential gene expression patterns were combined with promoter *cis*-acting element analysis to provide further understanding of flower development in response to moderate and severe drought stresses. We propose that many genes important for flower development are responsive to drought to protect reproductive success to some extent under drought stress.

## Results

### Morphological changes of *Arabidopsis* flowers in response to drought of different severities

**Figure 1:**
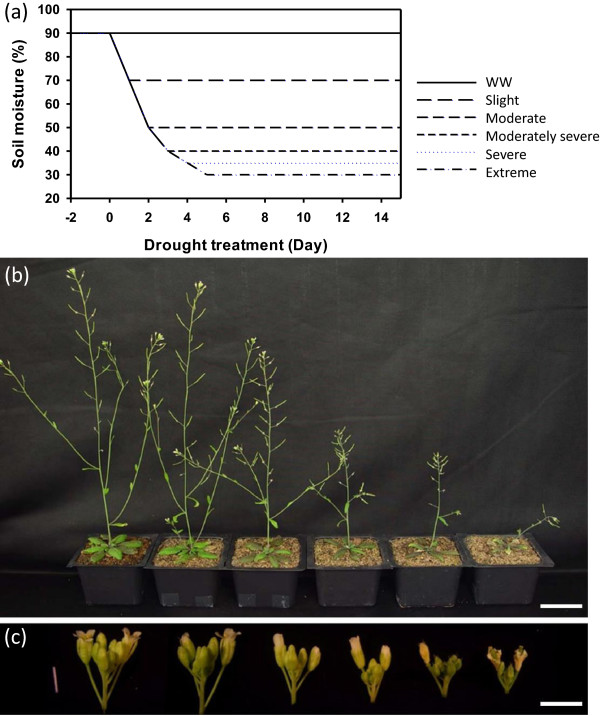
**Experimental design of different drought severities and overview of phenotypical changes of plant under drought. (a)** Time course of soil moisture measurement during the drought treatment, the x-axis indicates the start and the duration of drought. **(b) **Whole plants were photographed after ten days of treatment under six conditions (from left to right: Well-watered, Slight drought, Moderate drought, Moderately severe drought, Severe drought, Extreme drought). **(c)** Inflorescences dissected from the plants in panel b were photographed. Bars represent 5 cm in **(b) **and 3 mm in** (c)**.

To focus on the effects of drought treatment on reproductive development, we sowed one seed in each pot and allowed the plants to grow under well-watered conditions (90% water content) until bolting stage (about 24 days after planting), when the plants had an average height of about 1 cm and 8–9 rosette leaves. To investigate the impacts of different drought intensities on inflorescences, we divided the plants into six groups. One group (the control) was maintained throughout the experiment at 85-90% soil moisture by daily watering, whereas watering of the other five groups was stopped starting at the same time and until their soil moisture reached 70%, 50%, 40%, 35%, and 30% respectively. These five groups were then watered daily with an appropriate amount of water after weighing to maintain the soil moisture, respectively, at 65-70% (Slight), 45-50% (Moderate), 35-40% (Moderately severe), 30-35% (Severe), 25-30% (Extreme) (Figure 1a). Plants could not survive when soil moisture was lower than 25%. At 10 days after the initial drought treatment, the plant height showed varying degrees of reduction depending on the severity of drought treatment, from very slight to extremely severe (Figure 1b).

**Figure 2:**
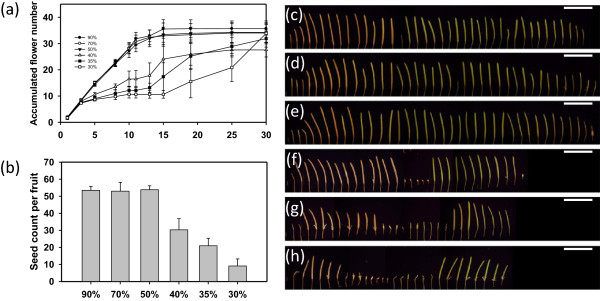
**Characterization of reproductive development under different drought severities. (a)** The accumulated flower numbers were affected by drought stress; **(b)** The seeds count per silique on main stems under different drought severities; **(c)**-**(h)** Drought stress affected the number of siliques, **(c)** Well-watered; **(d)** Slight drought; **(e)** Moderate drought; **(f)** Moderately severe drought; **(g)** Severe drought; **(h)** Extreme drought. Bar = 2 cm.

In addition, drought stresses also resulted in fewer branches, flower buds and siliques, especially when the water levels were low (Figure 1c). To further examine the effects of varying drought on reproductive development, we counted the number of accumulated flowers on the main stem under various drought treatments. As shown in Figure 2a, plants produced similar numbers of flowers on the main stem as the well-watered control, as long as the soil water content was above 45%, even though plants with 45-50% water had shorter heights due to reduced stem elongation (Figure 1b). However, when the soil moisture was below 40%, the cumulative flower number increased more slowly than normal, stopped increasing for a few days, and then resumed the slow rise (Figure 2a), indicating that new flowers formed at slower rates under these drought conditions and that there was a short period of a few days when no new flowers were produced. This was similar to our previous observations of severely drought treated plants (at 30-35% soil moisture; [11]); nevertheless the results here suggest that more severe drought caused both a greater reduction in the rate of new flower production and a longer delay in the resumption of new flower emergence. It is also quite amazing that the plants under severe drought could still sustain the production of new flowers, eventually reaching almost the same total flower number on the main stem as the control group, suggesting that the plants had acclimated to the drought conditions during the treatment period.

We had previously found that the reproductive yield was sensitive to severe drought conditions [11]. To learn the effects of moderate drought conditions, we counted the seed number per seedpod and found that plants could endure slight to moderate drought without obvious reduction in seed number on the main stem (Figure 2b). Similar to the trend for total flower number, there was hardly any evident difference between the three groups with soil moisture of 50% or greater. However, an obvious reduction of yield was observed when the water content reduced to 40%, and even more severe losses for the 25-30% and 30-35% of soil moisture conditions with less than 1/5 of the control for the extreme drought. Furthermore, different from those under moderate or slight drought conditions (45-50% or more; Figure 2c-2e), plants with less water stopped producing siliques for a few days, longer under more severe drought (Figure 2f-2h), consistent with the lack of new flowers on those plants, as described above. We also noticed that some of the plants under extreme drought did not survive till the end.

### Overview of transcriptome analyses of inflorescences under moderate drought condition

**Figure 3:**
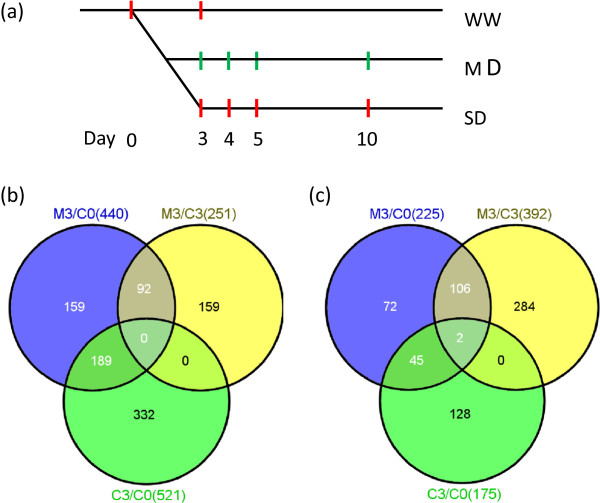
**Sample collection and transcriptomes from day 3 inflorescences to reveal plants early response to drought. (a)** Samples were collected at C0, C3 for WW plants and M3, M4, M5, M10 for MD plants. For comparison, T3, T4, T5, T10 for DT plants were from a previous study [11]. **(b)** A Venn diagram for up-regulated genes of M3 compared with C0, M3 compared with C3, and C3 compared with C0. **(c)** A Venn diagram down-regulated genes for the same comparisons as in **(b)**.

Based on the morphological observations, we hypothesized that *Arabidopsis* plants might adopt different mechanisms in response to moderate and severe drought conditions. Previously we showed that severe drought caused dramatic changes in the inflorescence transcriptome [11]. To further analyze the plant response to drought at the transcriptomic level, we analyzed the plants grown with the minimum soil moisture that they could acclimate with successful reproductive yield (45-50%) using the Affymetrix GeneChips, and we compared the gene expression with those of the well-watered control and also with the previous results under the severe drought condition (30-35%) [11]. To understand the temporal expression pattern, samples from wild-type inflorescences in *Arabidopsis* under different water conditions (50% and 90% water of the total dry soil weight) were collected at a series of time points (after 0, 3, 4, 5 and 10 days drought stress), samples from Control group were named as C0, C3, and M3, M4, M5, M10 for moderately drought group (Figure 3a). The previously analyzed severe drought samples were S3, S4, S5, S10 (Figure 3a) [11].

For each condition, we had at least two biological replicates for each time point and all the results were highly reproducible (all Pearson correlation coefficients > 0.98; Additional file 1). To focus on the genes significantly changed under drought compared with the well-watered condition, we only selected those whose expressions have: 1) more than two fold changes; 2) with q-values less than 0.01. According to these criteria, a total of 1830 genes were differentially expressed (up- and down-regulated) between the moderately drought at one or more of the four time points, Day 3, 4, 5 and 10 and the control group at C0 (Additional file 2). Specifically, 665 (M3/C0), 1049 (M4/C0), 1455 (M5/C0) and 659 (M10/C0) genes showed significantly differential expression at the respective time points (Additional file 3). Compared with C0, drought treated groups had increasing numbers of up-regulated genes during the early days of drought treatment, from 440 (at Day 3) up to 757 (at Day 4) and reaching the maximum at 1025 (at Day 5), but then decreased to 489 subsequently (at Day 10). A similar trend was found for the number genes that were significantly down-regulated under drought, increasing from 225 at Day 3 to 292 (Day 4) and 430 (Day 5), and then decreasing to 170 genes at day 10. Our results indicated that moderate drought induced altered expression of many genes in developing flowers, even though the morphology of these flowers seemed normal under such conditions.

### Early responsive genes to moderate drought function in multiple stress responsive pathways

**Figure 4:**
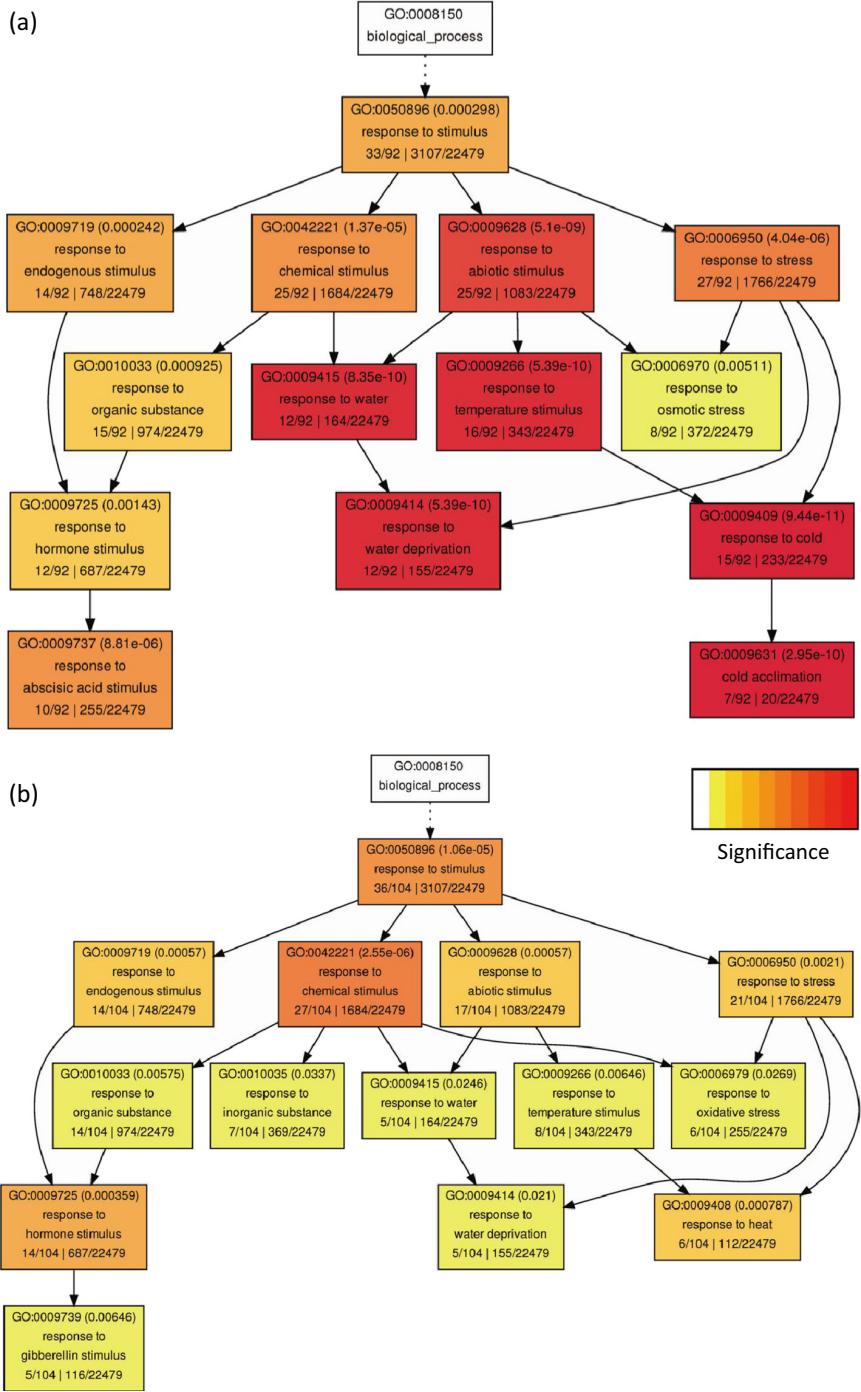
**GO enrichment analysis of the gene group that differentially expressed in both severe and moderate drought condition. (a)** The list of genes obtained from the overlapping gene group in both M3/C0 and M3/C3 in Figure 3b. **(b)** The list of genes from the overlapping gene group in both M3/C0 and M3/C3 in Figure 3c. Significance bar represents p-value from 1 × 10^-1^ to 1 × 10^-10^. P-values are shown in bracket of each box and gene count of each group is also included in the bottom line of the same box.

To better understand the plant early response to moderate drought, we further analyzed the transcriptomes on Day 3, by which time the soil moisture reached 45%-50%. Because the plants continued to grow during drought treatment, we reasoned that some gene expression changes were due to developmental regulation, among the 440 and 225 genes up-regulated and down-regulated, respectively, between moderately drought at day 3 (M3) and control at day 0 (C0) (Figure 3b and e3c, Additional file 4 and 5). To exclude the genes whose expression shifts were primarily due to developmental changes, we further compared gene expression between Day 3 to Day 0 in the control group (C3/C0) and found that 189 of the 440 up-regulated genes had differential expression and thus were identified as putative developmental genes. We further compared the drought-treated plants at Day 3 with the control C3 and saw that 251 genes were significantly up-regulated. Among the two sets of up-regulated genes from the M3/C0 and M3/C3 comparisons, 92 were common in both groups and thus considered as genes induced by moderate drought. Among the 92 genes induced at Day 3 are known genes involved in the plant response to water-deprivation, cold, salt and abscisic acid (ABA) stimulus (Figure 4a), consistent with the fact that these stresses lead to cellular water loss and that ABA is important for response to dehydration. We also examined the genes repressed under moderately drought condition within the first three days and found 106 genes as down-regulated in both comparisons (M3/C0 and M3/C3), but not in the C3/C0 comparison (Figure 3c, Additional file 5). The GO analysis showed that genes responsive to gibberellin, heat, oxidative stress and chemical stimulus were enriched (Figure 4b).

### Comparison between genes responsive to moderate and severe drought

**Figure 5:**
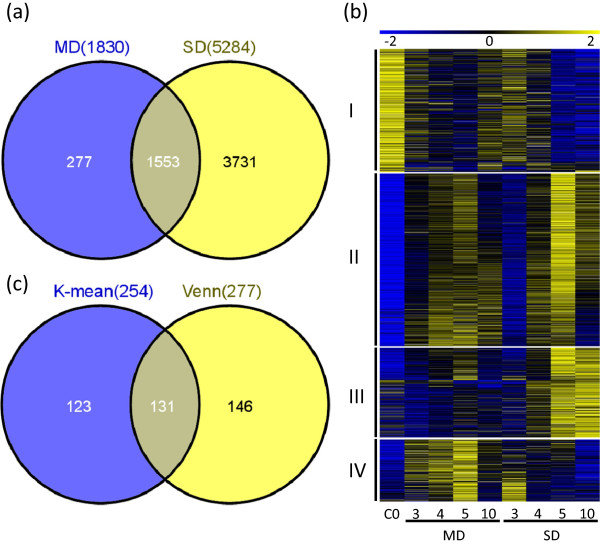
**Comparison of transcriptomes of inflorescence under moderate drought and severe drought. (a)** Venn diagram analysis showed the comparison on the differential expressed genes from moderate and severe drought treated plants. **(b)** K-means clustering of differentially expressed genes (1830) of moderate drought condition. The number indicates the fold change based on the normalized values of the hybridization signals in log2 format between one of the drought-treated groups and the control group (C0). **(c)** Comparison of the genes in the cluster IV of panel b (254 genes) with the genes specifically differentially expressed in moderate drought (277 genes in panel a).

We had previously compared transcriptomes of inflorescences under severe drought condition (30-35%) at Day 3, 4, 5 and 10 with those of control group [11] and found 5284 genes showing differential expression (Additional file 6). Among those genes, 1553 genes were differentially expressed under both drought conditions, and 277 genes were differentially expressed only under moderate drought (Figure 5a, Additional file 7). Preliminary hierarchical clustering suggested that there were four clusters; we then applied K-means method to cluster all 1830 genes into four clusters (Additional file 1). As shown in Figure 5b, genes in each cluster have distinct expression patterns. For cluster I, the highest expression level was observed at C0 (Day 0 without any treatment), suggesting that they function in early inflorescence development but were repressed at both drought conditions. Those genes in cluster II were induced by both drought treatments and with a greater extent at Day 5, suggesting their possible roles under both intensities of drought stress. The higher levels of induction at Day 5 under severe drought further suggested that they might be more active under such conditions. Furthermore, genes in cluster III had sustained high levels of expression under the severe drought but no obvious induction under moderate drought, suggesting that their function might not be active under moderate drought. Interestingly, the genes in the last group had an opposite expression pattern compared with cluster III: with comparatively high levels of expression under moderate drought condition. These last two clusters of genes strongly suggested that the molecular and physiological responses to moderate and severe drought conditions are not just quantitatively different in terms of number of genes induced or levels of expression changes, but also qualitatively distinctive in terms of the sets of genes induced. The presence of genes preferentially induced by moderate drought specifically points to distinct programs the plant uses in response to moderate water shortage.

To compare putative gene functions between these clusters, we examined the GO categories for the four clusters above. In general, genes responsive to stimulus and pollen tube growth were enriched in all clusters, consistent with their expression changes under severe drought condition [11]. In cluster I, gene functions in nucleosome assembly and response to GA and SA were enriched, suggesting that the observed decrease in stem elongation under moderate drought (Figure 1b) could be due to reduction of nucleosome assembly with possible effect on transcription, and that the reduced expression of genes for the GA signaling pathway was consistent with the fact that GA is important for stem elongation [23]. The reduced number of SA signaling genes suggests that plants under moderate drought might be more susceptible to diseases. Genes in cluster II were enriched for functioning in pollen tube growth and response to water deprivation, suggesting that their elevated expression under both drought conditions were important and might be responsible for the nearly normal reproductive development under moderate drought. Regulatory genes including those controlling transcription, response to hormones including ABA, GA, JA, ET, SA, IAA, and water deprivation were enriched in the 3nd cluster, suggesting that more severe drought caused greater changes in the transcriptome in part by elevating the activities of transcriptional regulators and by strengthening hormone signaling. Interestingly, genes annotated with function in photosynthesis, pigment biosynthesis and response to red light were enriched in cluster IV, whose expression levels were higher under moderate drought condition than under severe drought condition. This suggests that the nearly normal development of these plants might have been facilitated by the enhanced functions of these genes.

To investigate further what genes might be important for maximal reproductive yield under moderate drought with almost normal morphology, we compared the 277 gene that were only differentially expressed under moderate drought but not under severe drought, with the 254 genes in cluster IV of the above K-means clustering analysis. We found that 131 genes were shared between both groups (Figure 5c, Additional file 7), including 8 transcription factors (Table 1). Among these, *NF-Y2*, *NF-Y8* and *NF-Y10* were previously reported as regulators involved in photosynthesis and drought responses [24-26]. Another gene highly induced by moderate drought, *SOC1,* is a positive regulator of flowering downstream of *FT*[27,28]. *MYB11*, known as a member of the R2R3 factor gene family functioning essentially in flavonol glycoside accumulation [29], was also induced greatly by moderate drought but not by severe drought. The increased expression levels of these genes suggest that they could be important for flowering and photosynthesis to ensure reproductive success under moderate drought.

**Table 1:** Transcription factors showed induction in flowers specifically under moderate not severe drought condition

**AGI**	**Description**	**C0**	**M3**	**M4**	**M5**	**M10**	**S3**	**S4**	**S5**	**S10**
AT3G05690	NF-YA2	5.71	7.61	7.55	7.66	6.25	6.42	6.04	5.92	6.23
AT1G17590	NF-YA8	5.35	6.62	6.62	6.84	6.30	6.06	5.98	6.15	6.34
AT5G06510	NF-YA10	5.53	7.97	7.28	7.69	5.76	5.76	5.25	5.08	5.57
AT2G45660	SOC1	7.09	7.66	7.97	8.27	7.53	7.60	7.45	7.87	7.48
AT3G62610	MYB11	6.90	8.11	7.15	7.54	7.55	7.10	6.47	6.15	6.33
AT1G69570	Dof-type zinc finger TF	6.68	7.02	7.31	7.99	7.14	7.23	6.99	6.92	6.60
AT1G26610	C2H2-like zinc finger TF	7.34	7.88	8.20	8.41	8.01	7.69	8.17	7.93	7.52
AT5G50010	Sequence-specific TF	6.32	7.55	7.45	7.29	6.76	6.82	6.42	6.40	6.31

The list contains representative genes that were highly induced under moderate drought in flowers. All expression values are as log2 values; the same is true for the other tables.

### Enrichment of known *cis*-elements in the regulatory regions of differentially expressed genes

Studies of stress responsive genes have identified *cis*-elements for transcriptional regulation, such as the ABRE, MYBR and DRE motifs [1,22,30-32]. To test whether such motifs might be associated with genes that were differentially expressed in response to moderate drought, we searched for the known motifs in the putative promoter sequence (1 kb upstream of start codon) of all 1830 differentially expressed genes (Additional file 8). We found 274 genes with the ABRE site (1639 with the core motif ACGT), 1220 with the MYB binding site (WAACCA), as well as 242 with the DRE motif (RCCGAC) in the putative promoter sequences. In addition to these known binding motifs potentially involved in drought response, we also searched for other known *cis*-acting regulatory elements for members of transcription factor families: NAC family (1378 with its core binding motif: CACG), MYC or the bHLH family (1776 with canonical E-box: CANNTG and 457 with core motif G-box: CATGTG) and WRKY (346 with its binding site: TGACY). Besides, several known consensuses involved in transcriptional activation were also identified in the putative sequences, such as TATA-box and CAAT-box. Because the MYB, MYC, NAC and WRKY transcription factor families also include members that have functions distinct from response to environmental stresses, the presence of these *cis*-elements alone does not imply regulation by stress signals. Nevertheless, the combination of stress-induced expression and presence of related *cis*-elements makes a stronger case for such regulation.

### Genes for transcription factors were induced more by severe drought

We showed that the effect of drought on reproductive development was more drastic for severe drought than moderate drought. However, it is not known which genes are induced in a similar way, more under severe drought. By comparing the inflorescence transcriptome under severe and moderate drought conditions, we identified genes that were induced to a greater extent under severe drought, particularly in the 2nd cluster. From the GO results, we learned that transcription factors and transporters were among the enrich categories. We focused on the genes that have significantly more increased expression under severe drought compared with moderate drought, other than the genes that have preferential expression in moderate drought compared with well-watered condition (q < 0.01, two-fold change). At Day 3, no genes satisfying these criteria were found, but at later time points several genes with this expression pattern were identified (14 genes at Day 4, 62 genes at Day 5 and 26 genes at Day 10). This trend is consistent with our observation that the floral development resumed at Day 10 after a short pause following the initial drought treatment (Figure 1d).

Many genes within this group had important molecular functions, such as transcriptional regulation (Table 2). For example, genes for ATHB-7 and ATHB-12, members of the same phylogenetic clade γ with a homeodomain closely linked to a leucine zipper motif, showed preferential expression at Day 4, consistent with previous finding of drought or ABA induced expression in vegetative organs (root, leaf and stem) [33]. Two other genes encoding homeodomain factors ATHB-2 and ATHB-5 were also in this cluster, and phylogenic analysis using the homeodomains showed that they belong to a group δ closed to the one of ATHB-7 and ATHB-12 [34]. All four proteins belong to the same class Ι of HD zip proteins and have been shown to be responsive to ABA and salt stress at the seedling stage [34]. Therefore, it is possible they are also involved in drought stress response during flower development.

**Table 2:** Expression of genes known as transcription factors

**AGI**	**Description**	**C0**	**M3**	**M4**	**M5**	**M10**	**S3**	**S4**	**S5**	**S10**
At2g18550	ATHB-2/HB21	4.92	5.02	6.18	6.41	5.51	4.52	7.71	9.82	8.07
At4g36740	ATHB-5/HB40	4.23	4.52	5.47	6.03	5.63	4.83	7.60	9.85	9.74
At2g46680	ATHB-7	5.86	6.98	7.86	8.93	6.83	6.74	9.42	11.5	10.7
At3g61890	ATHB-12	5.37	6.44	7.30	9.18	6.13	7.56	8.95	11.3	11.2
At1g52890	NAC19	7.12	9.34	9.93	9.57	9.08	8.48	10.6	11.4	9.79
At3g04070	NAC47	5.08	5.63	5.93	6.45	5.27	4.99	6.84	8.81	6.73
At5g39610	NAC92/CUC2	5.16	6.02	6.37	6.68	6.16	5.94	6.64	7.74	5.64
At2g19810	OZF1	8.44	8.94	9.35	9.69	8.68	8.84	9.75	10.7	11.4
At4g29190	OZF2	7.65	8.25	8.85	9.34	8.29	8.48	9.69	11.6	11.5
At3g24520	HSF1	5.18	5.96	6.43	6.64	6.30	5.99	7.97	9.50	10.2
At1g76590	PLATZ TF	5.58	6.48	7.35	6.98	5.71	5.27	8.38	10.7	10.7

Several transcription factor genes in this cluster are members of the NAC family, with 102 genes in *Arabidopsis*[35]. Three NAC genes were induced by severe drought, including ANAC92, which belongs to the NAM clade. ANAC92 is known to function in the formation and development of the shoot apical meristem (SAM), and is redundant with CUC1 [36]. Another study suggested that ANAC92 regulates senescence in response to salt by controlling several downstream genes in a stage dependent way [37], similar to what we have observed in this drought study. The other two NAC members are ANAC19 and ANAC47, both members of the AtNAC3 group. Previous studies support roles of NAC proteins in stress response in *Arabidopsis* and rice [11,25].

Other transcription factors in this cluster included HSF1, PLATZ, OZF1 and OZF2. The OZFs are the closely related with two CCCH motifs [38]. Both OZF factors are ABA-responsive and OZF2 is involved in the ABI2-mediated signaling pathway [39,40]. It is possible that the two OZF function redundantly to assist the plant in response to various stresses. HSF1 is involved in response to a combination of drought and heat stress but more thorough experimental confirmation is needed [41].

### Moderate drought induced genes for transport, ABA-dependent pathway and reproduction

In addition to the transcription factors, many other genes also showed expression alteration responsive to moderate drought. Not surprisingly, many genes encoding transporters had elevated expression levels (Table 3). We also found that genes involved in male reproduction, late embryogenesis and seed dormancy were activated. Genes encoding four late embryogenesis abundant (LEA) proteins that protect other proteins from desiccation were in this group and some of them are known to respond to drought (Table 4) [41]. Interestingly, a gene called *MATERNAL EFFECT EMBRYO ARREST 25* (*MEE25*) coding for a UDP-glucose 4-epimerase was also in this group and it was suggested to function in male reproductive development [42]. Two other male reproductive genes were also found in this group of genes induced by moderate drought but not severe drought, partially explaining the delayed impact of drought on flowering observed in previous morphological analysis of plants under severe drought [11]. The identification of these functional genes suggested that both drought avoidance genes and drought tolerance genes were involved in the differential response of inflorescence to drought stress of different extent.

**Table 3:** Expression of genes involved in transportation

**AGI**	**Description**	**C0**	**M3**	**M4**	**M5**	**M10**	**S3**	**S4**	**S5**	**S10**
At1g02390	Acyltransferase2	4.84	5.32	5.61	6.32	5.81	5.44	6.34	7.85	7.79
At5g26340	Hexose transporter	6.35	8.17	8.69	7.54	7.96	6.23	7.91	8.85	8.47
At4g35190	Decarboxylase	7.56	9.18	9.37	9.85	9.27	8.35	10.0	10.9	9.66
At3g43270	Pectinesterase	6.71	8.54	8.84	8.86	8.78	7.89	9.04	9.58	9.25
At1g32450	PTR2-B	7.24	8.70	9.15	9.18	8.70	8.29	8.93	10.2	9.23
At5g47560	Dicarboxylate cotransporter	7.29	8.22	8.77	9.59	8.81	8.50	9.28	10.6	10.4
At1g78070	Transducin	5.70	6.37	6.50	7.19	5.96	6.34	6.93	8.66	8.17
At2g41190	Amino acid transporter	7.24	7.75	8.26	8.69	7.70	7.52	9.64	11.9	11.6
At5g01520	Zinc ion binding	6.18	6.81	7.39	8.08	6.85	6.04	8.62	10.4	9.53

**Table 4:** Expression of genes involved in embryogenesis and reproductive development

**AGI**	**Description**	**C0**	**M3**	**M4**	**M5**	**M10**	**S3**	**S4**	**S5**	**S10**
At1g01470	LEA14	7.55	8.44	8.91	8.86	8.71	8.17	9.54	10.8	10.8
At2g35300	LEA18	4.46	6.03	6.59	7.49	6.74	5.32	8.62	10.7	10.1
At5g06760	LEA4-5	6.69	7.49	8.09	8.69	8.40	7.38	9.74	11.9	11.8
At1g52690	LEA 7	4.61	6.37	8.66	10.1	7.86	7.88	11.9	13.8	13.4
At1g64110	DUO1-activated ATPase 1	4.43	4.96	5.87	6.67	5.37	4.68	7.57	10.1	9.95
At2g34850	MEE25	6.14	7.25	7.43	7.65	6.78	6.95	7.36	8.83	9.53
At4g14020	Pollen tube	5.17	6.35	6.45	6.64	5.59	6.15	7.54	8.82	7.97
At2g37870	Seed storage 2S	6.13	6.22	7.85	8.72	6.77	6.27	9.36	11.0	11.6

The crosstalk between multiple pathways in response to different stresses has been shown in many studies on vegetative development [41]. It is not surprising that known genes involved in known stress responsive pathways were also in this group (Table 5). The ABA signaling pathway is one of the key mechanisms important for response to drought stress in diverse plant groups [16,43,44]. In our study, we also found eight genes in the ABA signaling pathway with increasing expression levels as the drought severity became more intensive. Besides, genes responsive to cold and salt stresses were also found in our study, suggesting that crosstalk between different pathways also exists in the inflorescence.

**Table 5:** Expression of genes involved in known stress responsive genes

**AGI**	**Description**	**C0**	**M3**	**M4**	**M5**	**M10**	**S3**	**S4**	**S5**	**S10**
At5g57050	ABI2	7.14	7.60	8.24	8.41	7.78	7.54	8.81	10.4	10.3
At1g69260	AFP1	5.95	6.51	7.15	8.12	7.28	7.35	8.52	10.1	10.5
At3g29575	AFP3	8.11	8.6	9.22	9.42	9.37	8.76	9.82	10.8	10.8
At5g66400	ATDI8	6.44	6.92	8.95	10.6	7.64	7.86	12.2	14.1	14.1
At5g64260	Exordium like 2	9.45	9.53	10.2	10.7	9.84	9.60	11.4	12.8	12.5
At5g59220	PP2C GENE 1	8.06	8.78	9.47	9.80	9.34	9.02	10.6	12.1	12.2
At1g07430	PP2C GENE 2	6.17	8.97	9.49	9.19	8.31	7.64	9.08	10.9	10.8
At3g11410	PP2CA	8.79	9.85	10.2	10.4	10.1	9.72	11.0	12.2	12.0
At3g50970	LTI30-cold	4.72	9.34	10.3	9.96	6.85	5.94	9.56	11.5	11.3
At4g30960	CIPK6-salt	8.84	10.4	10.5	10.8	10.3	9.72	10.8	11.9	12.0
At5g02020	Salt induced serine rich	6.61	6.97	7.99	9.16	7.93	7.02	10.4	12.3	11.9

### Genes activated by moderate drought but not by severe drought

**Figure 6:**
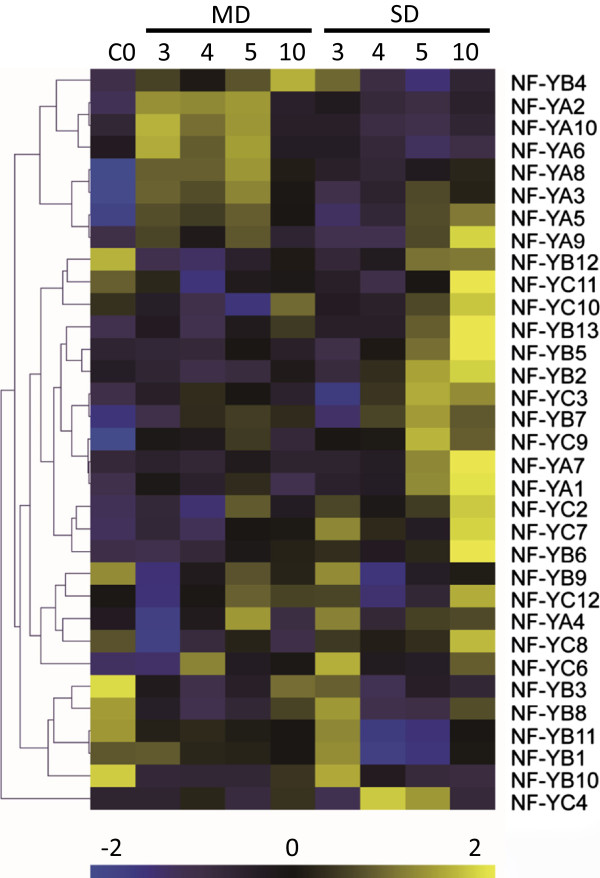
**Hierarchical clustering of NF-Y gene family under drought condition.** Yellow color represents high expression while blue color represents low expression. Hierarchical clustering was performed on transcripts ratios of all conditions. C represents control: well-watered group; MD represents moderate drought; SD represents severe drought.

As mentioned in the morphological analysis, there is no obvious reduction of yield on the main stem under moderate drought condition (50%) but there was a significant loss in severe drought (35%) (Figure 1, [11]). It is possible this is in part due to some of the genes that were induced under moderate but not severe drought. From our microarray data, we observed that genes in the third cluster shared a similar expression pattern that reached the highest expression levels under moderate drought. Among the 55 genes with elevated expression in MD that the control and SD on day 5 using stringent criteria (2 fold changes, q < 0.01), were four genes encoding Nuclear Factor Y transcription factor subunits (NY-Fs, also known as CCAAT-bind factors) (Figure 6). In *Arabidopsis* has 36 genes for CCAAT-bind factors (10 NF-YA, 13 NF-YB and 13 NF-YC) and they are thought to act as heterotrimers. After the first identification of CCAAT-binding factors in *Arabidopsis*[45], a few studies have reported about their functions in the development process especially in flowering [24,46]; the adaption to various stresses, including drought, osmotic stress and nutrient deprivation [47,48]; and the response to plant hormones, such as ABA [49]. We further investigated the expression of all the genes of NF-Y family and found that seven of the ten NF-YA were up-regulated under moderate drought (Figure 6). The hierarchical clustering on the basis of their expression levels suggested that the NF-YA subgroup is more responsive to moderate drought. It is possible that under moderate water stress NF-YA (CCAAT-binding factors) are activated to maintain reproductive growth.

### Signal pathways in response to moderate drought and severe drought

As mentioned above, 5284 genes were identified as differentially expressed under the SD condition and only 1830 genes under the MD condition. Among those genes, 1553 genes were detected in both studies. Using AgriGO software, we found different GO categories were enriched, including transcription regulator, transporter, enzyme and catalytic activity. The enrichment of similar categories was also observed in the group only differentially expressed under SD condition compared with control, except for the GO category of “binding activity” (Figure 4). This result indicated that the mechanisms plant employ to cope with varying levels of dehydration might be very similar.

85 transcription factors were differentially expressed under both MD and SD conditions, including members of DREB, NAC, AP2/ERF, MYB, bZIP, PLATZ, homeodomain, WRKY, zinc finger and HSF gene families. In addition to gene families mentioned above, genes in the AGL and BEH families were also identified in this category. The AGL family is commonly involved in the floral developmental process, thus it is consistent with our observation that only severe drought but not moderate drought, significantly influences the essential developmental process and cause loss of yield.

To find putative transcriptional regulatory network in response to drought, we investigated the putative promoter sequences of genes differentially expressed under drought condition. We searched for more than one hundred known binding motifs (from PLANTCARE) in the four clusters. Interestingly, the ABRE and ABRE-like binding motif were enriched in the 2nd and 3rd clusters (both within the first 0.5 kb and 1 kb). It is not surprising that ABA-independent pathway is very important in both moderate and severe drought response. Other binding motifs, such as E-box and G-box, were also enriched in these two clusters, suggesting putative transcription factors, such as those in the bHLH family, controlling some genes in the two clusters. The enrichment of *cis*-regulatory elements is not as significant when we searched in longer sequence (3 kb upstream of genes in each cluster). Though the NF-Y family members were enriched in the 2nd cluster, the binding site of NF-Y was not obviously enriched in any clusters using different length of putative promoter sequence. However, we still find many genes in this cluster with the CCAAT motif.

## Discussion

Inflorescence development is one of the essential constraint factors affecting plant yield. In this study, a moderate drought condition was applied to examine the inflorescence transcriptome to identify the gene activities that plant uses in response to drought, in a way similar to the recent study about the transcriptome analyses on inflorescence under severe drought condition [11]. Although the effects of drought on reproductive development cannot fully be understood at this time and even vegetative organs may also play vital roles in success or failure to seed generation, the comparison of transcriptomes under two different water deficit conditions still can provide us a better understanding of not only the regulatory network in response to drought stress in flowers but also the different strategies that plants use to acclimate to different drought severities.

### Reproductive acclimation to different drought conditions

Under moderate drought condition, *Arabidopsis* was able to achieve normal seed production on the main stem, similar to plants under well-watered condition, though there might be difference in seed contents and germination ability. The normal reproductive capacity of the main inflorescence indicates that the plant can maximize the use of limited water resources to ensure the production of the next generation even in an unfavorable environment. Similar observation was made in the study of *Allocasuarina luehmannii* whose vegetative growth appear to be normal under moderate drought, but not severe drought [50]. In Geranium, moderate drought did not affect the overall quality of plants, but severe drought caused a reduction in the number of flowers per plant [51].

The sterile siliques found on the main stem of the plant grown under moderately severe, severe, and extreme drought conditions might be an important strategy for the plant to survive more extreme unfavorable environment. Under such extreme conditions, the sacrifice of a portion of the reproductive structures would limit the use of energy and water, allowing the precious resources to support the remaining reproduction for survival to the next generation. It is possible that alterations in the distribution of nitrogen and carbon assimilation to different plant parts to maintain reproductive ability are part of the response to drought, as reported before [52,53].

### Transcriptional reprogramming of inflorescence under moderate drought

Although the moderate drought did not cause dramatic morphological changes during reproductive development, a large number of genes (1830) were differentially expressed compared with those in well-watered plant. This was likely due to mechanisms that have evolved to protect plants against biotic and abiotic stresses without severe morphological changes, particularly to help plants to respond to mild environmental changes. Among these are the genes that function in response to stimulus especially ABA signaling and water deprivation and the genes that function in pollen tube growth, suggesting that these two aspects of drought response are critically important for plant growth under moderate drought conditions. One is accelerating the pollination and fertilization processes by activating those genes involving in pollen tube elongation; another aspect is promoting and strengthening the defense system to help plant to be more tolerant against drought stress. Therefore, it is likely that the reprogramming of the inflorescence transcriptome is at least in part important for the successful reproduction under moderate drought.

### Difference of transcriptomes in response to moderate drought and severe drought

Transcriptomic analyses indicated that both moderate and severe drought conditions induced dramatic responses during flower development. Nuclear factor Y (NF-Y) is composed of three distinct subunits (NF-YA, NF-YB, and NF-YC). Interestingly, majority of genes encoding NF-Ys were found induced more under severe drought than moderate drought. However, seven out of the ten genes of the NF-YA subfamily were found to be induced by moderate drought but that is not true under severe drought condition, suggesting their possible roles in early response to drought stress and low intensity of drought. For example, NF-YA 2, 6, 8 and 10 were hardly induced at all under severe drought, indicating their specific roles in moderate drought response. NF-YA 3, 5 and 9 could be induced 3 days after moderate drought treatment, however , under severe drought they were induced much later (after day 5) (Figure 6). NF-YB 2 and NF-YB 3 are known as flowering time regulators and can interact with the floral promoting protein CONSTANS (CO) in the photoperiod dependent flowering regulatory network, and NF-YBs were also reported to interact with MADS-box genes in rice using an *in vitro* assay [24,54]. NF-YA 5 and NF-YB 1 were reported to function in promoting drought resistance in *Arabidopsis*[26,55]. NF-YB 6 and NF-YB 9 control early embryogenesis and embryo development, and also involved in seed maturation in *Arabidopsis*[49,56]. In soybeans (*Glycine max* L.) GmNFYA3 is a positive regulator in drought response [57]. Additional experiments of NF-YAs in *Arabidopsis* suggested their roles in modulating gene regulation through positive and negative mechanisms [58]. Among the differentially expressed genes only due to moderate drought, we also identified that *SOC1/AGL20* were significantly up-regulated by moderate drought. This could be caused by induction of NF-Ys and also indicate that SOC1 could act as an important node that connects both reproductive development and stress response.

### Comparison with transcriptomes of vegetative tissues

To learn possible similarities and differences in gene activities affected by drought between reproductive and other tissues, we compared our results with other transcriptomes from vegetative tissues. Harb et al. studied transcriptome at vegetative stages in early response to soil drought condition in *Arabidopsis*[59] and found 2039 genes differentially expressed in response to moderate drought (30% soil capacity in Harb’s study) that is similar with 50% soil moisture in our study (Additional file 9). Among the 2039 genes, 372 were also differentially expressed in our data (1830 differentially expressed genes) (Additional file 10), including NF-Y2, NF-Y3, NF-Y5, NF-Y8 and NF-Y10, indicating that the NF-Y genes are important for response to moderate drought in both vegetative and reproductive organs in *Arabidopsis*. Among the 1458 genes that were differentially expressed only in our reproductive transcriptome but not in the Harb et al. study, the genes involved in response to stimulus such as ABA, GA, water deprivation and ROS are highly enriched (Additional file 9), suggesting that there might also be different regulatory pathway or genes functions in different tissue types in response to drought stress. Further efforts are needed to elucidate the mechanistic differences in response in different tissue types and to different drought severities.

## Conclusion

In conclusion, we observed that moderate drought did not cause dramatic reduction of reproductive yield, but did induce altered expression of many genes, although fewer than those under severe drought. A comparison of transcriptomes in response to moderate or severe drought, we discovered that the CCAAT-binding factors/NF-Ys were specifically induced by moderate drought and might have a specific function under this condition. Our results indicate that plants respond to mild water stress by inducing many genes, whose function are likely important in protecting plants against the stresses and in ensuring reproductive success under such conditions.

## Methods

### Plant materials

In this study, morphological analyses under different water conditions were performed on Col-0, which has been sequenced completely. Drought assay was done as described in our previous study with some minor changes [11]. Seeds were directly planted into pots containing 100 g soil consisting of dry soil (Metro-Mix 360, Sun Gro Horticulture Canada Ltd) and greens grade (Turface profile greens grade, Profile Products LLC) by a ratio 3:2 in volume. The water-holding capacity of 90 g (defined as 90% soil moisture, also indicated as 90% field capacity) as Well-watered condition was measure by weighing on a scale. 70g, 50g, 40g, 35g and 30 g of water, respectively, were added to the soil mixture to achieve different water-deficit conditions. After two days of seed stratification in dark at 4°C, all the plants were grown in growth chamber under normal growth condition (22°C, 16 h/8 h, day/night photoperiod, ~300 μmol m^-2^ s^-1^ photon flux, 60% humidity) until the plant had just begun to flower (bolting was visible with a main inflorescence stem of about 1 cm and unopened floral buds) when plants were subjected to different types of drought treatment when the main stem is about 1 cm high [11]. Plants for morphological analyses were then observed until almost all the siliques were matured and ready to be harvested (about 50 days after planting).

Samples collected for microarray were prepared as follow: the moderate drought (MD) and severe drought (SD) treatments started by withholding water. The relative soil moisture content reduced to the expected degree (MD: 50% and SD: 35%) three days after the starting point (C0). We maintained the soil water condition (30% - 35%, 45% - 50%, and 85% - 90% in control group) for 3, 4, 5 and 10 days (labeled as D 3, D 4, D 5 and D 10, started from water withholding day). Unopened flower samples were then collected, from both M and S drought treated groups and control groups. Two biological replicates from the inflorescences were collected at each time point from each group.

The condition of our drought assay was important to obtain reproducible results; different soil conditions or chemical treatments that mimic osmotic stress could result in different transcriptome changes.

### Microarray experiment

Following the Affymetrix GeneChip Expression Analysis Overview described on the website (http://www.affymetrix.com), cRNAas were synthesized for hybridization as described [60]. Hybridization, washing, staining, scanning and data collection were performed in Genomics Core Facility at Pennsylvania State University.

### Microarray analysis

Normalization was applied using Bioconductor package in R by RMA, and all the expression values were converted to logarithms base 2. We then used LIMMA package to compare signals from control and well-watered inflorescences. Only genes with more than two-fold changes were selected in addition to the statistical criterion: Q-value (FDR) less than 0.01.

K-means clustering of co-expressed genes was performed by MeV 4.9 [61]. The normalized values of hybridization signal in log2 were used in K-means analysis, and the heatmap was generated base on the difference from the mean of the values of each gene. For the identification of the functions of the differentially expressed genes, the annotations of genes on ATH1 microarray chip were downloaded from Affymetrix website and we used the GO categorization function on TAIR website. To verify whether one category is enriched compared with the whole genome, we applied hypergeometric test and only the categories with p-value less than 0.05 were called statistically enriched group.

### *cis*-regulatory element analysis and GO analysis

Possible promoter sequences of all genes on the microarray chip (1 kb upstream of the start codon) were obtained from TAIR website. The numbers of binding sites of different transcriptional regulators were then counted. The identification of *cis*-regulatory binding site was conducted by perl [62]. The binding motifs were obtained from Gene Regulation and PlantCARE [63]. The Gene Ontology (GO) analysis was done by the agriGO software [64]. Significance bar represents p-value from 1 × 10^-1^ to 1 × 10^-10^.

### Availability of supporting data

The raw data sets supporting the results of this article are available in the Gene Expression Omnibus (GEO) repository under accession No GSE55431 (http://www.ncbi.nlm.nih.gov/geo/query/acc.cgi?acc=GSE55431). The data and analyses of transcriptomes under serve drought are available in GEO with accession No GSE40998 (http://www.ncbi.nlm.nih.gov/geo/query/acc.cgi?acc=GSE40998) [11].

## Abbreviations

MD: Moderate drought; SD: Severe drought.

## Competing interests

The authors declare that they have no competing interests.

## Authors’ contributions

HM and ZS designed the research and supervised all the experiments. ZS and NLS performed the drought experiments. XM and ZS analyzed the microarray data and interpreted the analyzed results. XM, ZS and HM discussed the results and wrote the manuscript. All authors read and approved the final manuscript.

## Supplementary Material

Additional file 1 Microarray data correlation between two biological replicates at each time point. Click here for file

Additional file 2 The list of genes that were differentially expressed in any of the four time points, Day 3, 4, 5 and 10 under moderate condition compared with control group C0. Click here for file

Additional file 3 The list of genes in each group of venn diagram analysis on up-regulated genes in M3, C3, C0. Click here for file

Additional file 4 The list of genes in each group of venn diagram analysis on down-regulated genes in M3, C3, C0. Click here for file

Additional file 5 The list of genes that were differentially expressed in any of the four time points, Day 3, 4, 5 and 10 under severe drought condition compared with control group C0. Click here for file

Additional file 6 The list of genes in each group of venn diagram analysis on differentially expressed genes in moderate and severe drought. Click here for file

Additional file 7 The list of genes in each group of venn diagram analysis on differentially expressed genes in moderate and severe drought. Click here for file

Additional file 8 Statistical Analysis of cis-Regulatory Element within 1kb Promoters Region of Differentially Expressed Transcription Factors. Click here for file

Additional file 9 Venn diagram and GO enrichment analyses of the comparison between our differentially expressed genes with the DE genes from vegetative tissue study by Harb et al. Click here for file

Additional file 10 The list of 372 genes differentially expressed in both vegetative transcriptome and our reproductive transcriptome under moderate drought. Click here for file

## References

[B1] Golldack D Luking I Yang O Plant tolerance to drought and salinity: stress regulating transcription factors and their functional significance in the cellular transcriptional network Plant Cell Rep 2011 30 8 1383 1391 10.1007/s00299-011-1068-0 21476089

[B2] Blum A Drought resistance, water-use efficiency, and yield potential - are they compatible, dissonant, or mutually exclusive? Aust J Agr Res 2005 56 11 1159 1168 10.1071/AR05069

[B3] Blum A Improving wheat grain filling under stress by stem reserve mobilisation Euphytica 1998 100 1–3 77 83

[B4] Finkelstein RR Gampala SSL Rock CD Abscisic acid signaling in seeds and seedlings Plant Cell 2002 14 S15 S45 12045268 10.1105/tpc.010441 PMC151246

[B5] van der Weele CM Spollen WG Sharp RE Baskin TI Growth of *Arabidopsis thaliana* seedlings under water deficit studied by control of water potential in nutrient-agar media J Exp Bot 2000 51 350 1555 1562 10.1093/jexbot/51.350.1555 11006306

[B6] Xiong L Wang RG Mao G Koczan JM Identification of drought tolerance determinants by genetic analysis of root response to drought stress and abscisic acid Plant Physiol 2006 142 3 1065 1074 10.1104/pp.106.084632 16963523 PMC1630748

[B7] Bohnert HJ Nelson DE Jensen RG Adaptations to environmental stresses Plant Cell 1995 7 7 1099 1111 10.1105/tpc.7.7.1099 12242400 PMC160917

[B8] Luan S Signalling drought in guard cells Plant Cell Environ 2002 25 2 229 237 10.1046/j.1365-3040.2002.00758.x 11841666

[B9] Yaish MW Colasanti J Rothstein SJ The role of epigenetic processes in controlling flowering time in plants exposed to stress J Exp Bot 2011 62 11 3727 3735 10.1093/jxb/err177 21633082

[B10] Jin Y Yang H Wei Z Ma H Ge X Rice male development under drought stress: phenotypic changes and stage-dependent transcriptomic reprogramming Mol Plant 2013 6 5 1630 1645 10.1093/mp/sst067 23604203

[B11] Su Z Ma X Guo H Sukiran NL Guo B Assmann SM Ma H Flower development under drought stress: morphological and transcriptomic analyses reveal acute responses and long-term acclimation in *Arabidopsis* Plant Cell 2013 25 10 3785 3807 10.1105/tpc.113.115428 24179129 PMC3877795

[B12] Ali M Jensen CR Mogensen VO Andersen MN Henson IE Root signalling and osmotic adjustment during intermittent soil drying sustain grain yield of field grown wheat Field Crop Res 1999 62 1 35 52 10.1016/S0378-4290(99)00003-9

[B13] Gordon LJ Finlayson CM Falkenmark M Managing water in agriculture for food production and other ecosystem services Agr Water Manage 2010 97 4 512 519 10.1016/j.agwat.2009.03.017

[B14] Jia HF Ma HT Wei MJ Calculation of the minimum ecological water requirement of an urban river system and its deployment: a case study in Beijing central region Ecol Model 2011 222 17 3271 3276 10.1016/j.ecolmodel.2011.05.026

[B15] Seki M Umezawa T Urano K Shinozaki K Regulatory metabolic networks in drought stress responses Curr Opin Plant Biol 2007 10 3 296 302 10.1016/j.pbi.2007.04.014 17468040

[B16] Hauser F Waadt R Schroeder JI Evolution of abscisic acid synthesis and signaling mechanisms Curr Biol 2011 21 9 R346 R355 10.1016/j.cub.2011.03.015 21549957 PMC3119208

[B17] Lippold F Sanchez DH Musialak M Schlereth A Scheible WR Hincha DK Udvardi MK AtMyb41 regulates transcriptional and metabolic responses to osmotic stress in Arabidopsis Plant Physiol 2009 149 4 1761 1772 10.1104/pp.108.134874 19211694 PMC2663747

[B18] Hossain MA Lee Y Cho JI Ahn CH Lee SK Jeon JS Kang H Lee CH An G Park PB The bZIP transcription factor OsABF1 is an ABA responsive element binding factor that enhances abiotic stress signaling in rice Plant Mol Biol 2010 72 4–5 557 566 20039193 10.1007/s11103-009-9592-9

[B19] Yoshida T Fujita Y Sayama H Kidokoro S Maruyama K Mizoi J Shinozaki K Yamaguchi-Shinozaki K AREB1, AREB2, and ABF3 are master transcription factors that cooperatively regulate ABRE-dependent ABA signaling involved in drought stress tolerance and require ABA for full activation Plant J 2010 61 4 672 685 10.1111/j.1365-313X.2009.04092.x 19947981

[B20] Kilian J Whitehead D Horak J Wanke D Weinl S Batistic O D'Angelo C Bornberg-Bauer E Kudla J Harter K The AtGenExpress global stress expression data set: protocols, evaluation and model data analysis of UV-B light, drought and cold stress responses Plant J 2007 50 2 347 363 10.1111/j.1365-313X.2007.03052.x 17376166

[B21] Sreenivasulu N Sunkar R Wobus U Strickert M Array platforms and bioinformatics tools for the analysis of plant transcriptome in response to abiotic stress Methods Mol Biol 2010 639 71 93 10.1007/978-1-60761-702-0_5 20387041

[B22] Rabbani MA Maruyama K Abe H Khan MA Katsura K Ito Y Yoshiwara K Seki M Shinozaki K Yamaguchi-Shinozaki K Monitoring expression profiles of rice genes under cold, drought, and high-salinity stresses and abscisic acid application using cDNA microarray and RNA gel-blot analyses Plant Physiol 2003 133 4 1755 1767 10.1104/pp.103.025742 14645724 PMC300730

[B23] Lee S Cheng H King KE Wang W He Y Hussain A Lo J Harberd NP Peng J Gibberellin regulates Arabidopsis seed germination via RGL2, a GAI/RGA-like gene whose expression is up-regulated following imbibition Genes Dev 2002 16 5 646 658 10.1101/gad.969002 11877383 PMC155355

[B24] Kumimoto RW Adam L Hymus GJ Repetti PP Reuber TL Marion CM Hempel FD Ratcliffe OJ The Nuclear Factor Y subunits NF-YB2 and NF-YB3 play additive roles in the promotion of flowering by inductive long-day photoperiods in Arabidopsis Planta 2008 228 5 709 723 10.1007/s00425-008-0773-6 18600346

[B25] Stephenson TJ McIntyre CL Collet C Xue GP *TaNF-YC11*, one of the light-upregulated NF-YC members in *Triticum aestivum*, is co-regulated with photosynthesis-related genes Funct Integr Genomics 2010 10 2 265 276 10.1007/s10142-010-0158-3 20111976

[B26] Li WX Oono Y Zhu J He XJ Wu JM Iida K Lu XY Cui X Jin H Zhu JK The Arabidopsis NFYA5 transcription factor is regulated transcriptionally and posttranscriptionally to promote drought resistance Plant Cell 2008 20 8 2238 2251 10.1105/tpc.108.059444 18682547 PMC2553615

[B27] Searle I He Y Turck F Vincent C Fornara F Krober S Amasino RA Coupland G The transcription factor FLC confers a flowering response to vernalization by repressing meristem competence and systemic signaling in Arabidopsis Genes Dev 2006 20 7 898 912 10.1101/gad.373506 16600915 PMC1472290

[B28] Immink RG Pose D Ferrario S Ott F Kaufmann K Valentim FL de Folter S van der Wal F van Dijk AD Schmid M Angenent GC Characterization of SOC1’s central role in flowering by the identification of its upstream and downstream regulators Plant Physiol 2012 160 1 433 449 10.1104/pp.112.202614 22791302 PMC3440217

[B29] Stracke R Jahns O Keck M Tohge T Niehaus K Fernie AR Weisshaar B Analysis of production of Flavonol Glycosides-dependent flavonol glycoside accumulation in Arabidopsis thaliana plants reveals MYB11-, MYB12- and MYB111-independent flavonol glycoside accumulation New Phytol 2010 188 4 985 1000 10.1111/j.1469-8137.2010.03421.x 20731781

[B30] Seki M Narusaka M Ishida J Nanjo T Fujita M Oono Y Kamiya A Nakajima M Enju A Sakurai T Satou M Akiyama K Taji T Yamaguchi-Shinozaki K Carninci P Kawai J Hayashizaki Y Shinozaki K Monitoring the expression profiles of 7000 *Arabidopsis* genes under drought, cold and high-salinity stresses using a full-length cDNA microarray Plant J 2002 31 3 279 292 10.1046/j.1365-313X.2002.01359.x 12164808

[B31] Shinozaki K Yamaguchi-Shinozaki K Seki M Regulatory network of gene expression in the drought and cold stress responses Curr Opin Plant Biol 2003 6 5 410 417 10.1016/S1369-5266(03)00092-X 12972040

[B32] Huang GT Ma SL Bai LP Zhang L Ma H Jia P Liu J Zhong M Guo ZF Signal transduction during cold, salt, and drought stresses in plants Mol Biol Rep 2012 39 2 969 987 10.1007/s11033-011-0823-1 21573796

[B33] Lee YH Chun JY A new homeodomain-leucine zipper gene from *Arabidopsis thaliana* induced by water stress and abscisic acid treatment Plant Mol Biol 1998 37 2 377 384 10.1023/A:1006084305012 9617808

[B34] Henriksson E Olsson AS Johannesson H Johansson H Hanson J Engstrom P Soderman E Homeodomain leucine zipper class I genes in Arabidopsis: expression patterns and phylogenetic relationships Plant Physiol 2005 139 1 509 518 10.1104/pp.105.063461 16055682 PMC1203399

[B35] Ooka H Satoh K Doi K Nagata T Otomo Y Murakami K Matsubara K Osato N Kawai J Carninci P Hayashizaki Y Suzuki K Kojima K Takahara Y Yamamoto K Kikuchi S Comprehensive analysis of NAC family genes in *Oryza sativa* and *Arabidopsis thaliana* DNA Res 2003 10 6 239 247 10.1093/dnares/10.6.239 15029955

[B36] Takada S Hibara K Ishida T Tasaka M The *CUP-SHAPED COTYLEDON1* gene of *Arabidopsis* regulates shoot apical meristem formation Development 2001 128 7 1127 1135 11245578 10.1242/dev.128.7.1127

[B37] Balazadeh S Siddiqui H Allu AD Matallana-Ramirez LP Caldana C Mehrnia M Zanor MI Kohler B Mueller-Roeber B A gene regulatory network controlled by the NAC transcription factor ANAC092/AtNAC2/ORE1 during salt-promoted senescence Plant J 2010 62 2 250 264 10.1111/j.1365-313X.2010.04151.x 20113437

[B38] Wang D Guo Y Wu C Yang G Li Y Zheng C Genome-wide analysis of CCCH zinc finger family in Arabidopsis and rice BMC Genomics 2008 9 44 10.1186/1471-2164-9-44 18221561 PMC2267713

[B39] Sanchez JP Duque P Chua NH ABA activates ADPR cyclase and cADPR induces a subset of ABA-responsive genes in *Arabidopsis* Plant J 2004 38 3 381 395 10.1111/j.1365-313X.2004.02055.x 15086800

[B40] Huang P Ju HW Min JH Zhang X Chung JS Cheong HS Kim CS Molecular and physiological characterization of the *Arabidopsis thaliana* Oxidation-related Zinc Finger 2, a plasma membrane protein involved in ABA and salt stress response through the ABI2-mediated signaling pathway Plant Cell Physiol 2012 53 1 193 203 10.1093/pcp/pcr162 22121246

[B41] Rizhsky L Liang H Shuman J Shulaev V Davletova S Mittler R When defense pathways collide: the response of Arabidopsis to a combination of drought and heat stress Plant Physiol 2004 134 4 1683 1696 10.1104/pp.103.033431 15047901 PMC419842

[B42] Boavida LC Shuai B Yu HJ Pagnussat GC Sundaresan V McCormick S A collection of *Ds* insertional mutants associated with defects in male gametophyte development and function in *Arabidopsis thaliana* Genetics 2009 181 4 1369 1385 10.1534/genetics.108.090852 19237690 PMC2666506

[B43] Raghavendra AS Gonugunta VK Christmann A Grill E ABA perception and signalling Trends Plant Sci 2010 15 7 395 401 10.1016/j.tplants.2010.04.006 20493758

[B44] Fujita Y Fujita M Shinozaki K Yamaguchi-Shinozaki K ABA-mediated transcriptional regulation in response to osmotic stress in plants J Plant Res 2011 124 4 509 525 10.1007/s10265-011-0412-3 21416314

[B45] Edwards D Murray JA Smith AG Multiple genes encoding the conserved CCAAT-box transcription factor complex are expressed in Arabidopsis Plant Physiol 1998 117 3 1015 1022 10.1104/pp.117.3.1015 9662544 PMC34917

[B46] Cai X Ballif J Endo S Davis E Liang M Chen D DeWald D Kreps J Zhu T Wu Y A putative CCAAT-binding transcription factor is a regulator of flowering timing in Arabidopsis Plant Physiol 2007 145 1 98 105 10.1104/pp.107.102079 17631525 PMC1976580

[B47] Chen NZ Zhang XQ Wei PC Chen QJ Ren F Chen J Wang XC AtHAP3b plays a crucial role in the regulation of flowering time in *Arabidopsis* during osmotic stress J Biochem Mol Biol 2007 40 6 1083 1089 10.5483/BMBRep.2007.40.6.1083 18047807

[B48] Nilsson L Muller R Nielsen TH Dissecting the plant transcriptome and the regulatory responses to phosphate deprivation Physiol Plant 2010 139 2 129 143 10.1111/j.1399-3054.2010.01356.x 20113436

[B49] Yamamoto A Kagaya Y Toyoshima R Kagaya M Takeda S Hattori T Arabidopsis NF-YB subunits LEC1 and LEC1-LIKE activate transcription by interacting with seed-specific ABRE-binding factors Plant J 2009 58 5 843 856 10.1111/j.1365-313X.2009.03817.x 19207209

[B50] Posch S Bennett LT Photosynthesis, photochemistry and antioxidative defence in response to two drought severities and with re-watering in *Allocasuarina luehmannii* Plant Biol (Stuttg) 2009 11 Suppl 1 83 93 19778372 10.1111/j.1438-8677.2009.00245.x

[B51] Sanchez-Blanco MJ Alvarez S Navarro A Banon S Changes in leaf water relations, gas exchange, growth and flowering quality in potted geranium plants irrigated with different water regimes J Plant Physiol 2009 166 5 467 476 10.1016/j.jplph.2008.06.015 18778872

[B52] Chaves MM Pereira JS Maroco J Rodrigues ML Ricardo CP Osorio ML Carvalho I Faria T Pinheiro C How plants cope with water stress in the field: photosynthesis and growth Ann Bot 2002 89 Spec No 907 916 12102516 10.1093/aob/mcf105 PMC4233809

[B53] Pinheiro C Chaves MM Ricardo CP Alterations in carbon and nitrogen metabolism induced by water deficit in the stems and leaves of Lupinus albus L J Exp Bot 2001 52 358 1063 1070 10.1093/jexbot/52.358.1063 11432922

[B54] Masiero S Imbriano C Ravasio F Favaro R Pelucchi N Gorla MS Mantovani R Colombo L Kater MM Ternary complex formation between MADS-box transcription factors and the histone fold protein NF-YB J Biol Chem 2002 277 29 26429 26435 10.1074/jbc.M202546200 11971906

[B55] Nelson DE Repetti PP Adams TR Creelman RA Wu J Warner DC Anstrom DC Bensen RJ Castiglioni PP Donnarummo MG Hinchey BS Kumimoto RW Maszle DR Canales RD Krolikowski KA Dotson SB Gutterson N Ratcliffe OJ Heard JE Plant nuclear factor Y (NF-Y) B subunits confer drought tolerance and lead to improved corn yields on water-limited acres Proc Natl Acad Sci U S A 2007 104 42 16450 16455 10.1073/pnas.0707193104 17923671 PMC2034233

[B56] Junker A Monke G Rutten T Keilwagen J Seifert M Thi TM Renou JP Balzergue S Viehover P Hahnel U Ludwig-Muller J Altschmied L Conrad U Weisshaar B Baumlein H Elongation-related functions of Leafy Cotyledon1 during the development of Arabidopsis thaliana Plant J 2012 71 3 427 442 22429691 10.1111/j.1365-313X.2012.04999.x

[B57] Ni Z Hu Z Jiang Q Zhang H GmNFYA3, a target gene of miR169, is a positive regulator of plant tolerance to drought stress Plant Mol Biol 2013 82 1–2 113 129 23483290 10.1007/s11103-013-0040-5

[B58] Leyva-Gonzalez MA Ibarra-Laclette E Cruz-Ramirez A Herrera-Estrella L Functional and transcriptome analysis reveals an acclimatization strategy for abiotic stress tolerance mediated by Arabidopsis NF-YA family members PLoS One 2012 7 10 e48138 10.1371/journal.pone.0048138 23118940 PMC3485258

[B59] Harb A Krishnan A Ambavaram MM Pereira A Molecular and physiological analysis of drought stress in Arabidopsis reveals early responses leading to acclimation in plant growth Plant Physiol 2010 154 3 1254 1271 10.1104/pp.110.161752 20807999 PMC2971604

[B60] Wijeratne AJ Zhang W Sun Y Liu W Albert R Zheng Z Oppenheimer DG Zhao D Ma H Differential gene expression in Arabidopsis wild-type and mutant anthers: insights into anther cell differentiation and regulatory networks Plant J 2007 52 1 14 29 10.1111/j.1365-313X.2007.03217.x 17666023

[B61] Yang C Xu Z Song J Conner K Vizcay Barrena G Wilson ZA *Arabidopsis MYB26*/*MALE STERILE35* regulates secondary thickening in the endothecium and is essential for anther dehiscence Plant Cell 2007 19 2 534 548 10.1105/tpc.106.046391 17329564 PMC1867336

[B62] Jurgens G El-Kasmi F Pacher T Strompen G Stierhof YD Muller LM Koncz C Mayer U Arabidopsis SNARE protein SEC22 is essential for gametophyte development and maintenance of Golgi-stack integrity Plant J 2011 66 268 279 10.1111/j.1365-313X.2011.04487.x 21205036

[B63] Parenicova L de Folter S Kieffer M Horner DS Favalli C Busscher J Cook HE Ingram RM Kater MM Davies B Angenent GC Colombo L Molecular and phylogenetic analyses of the complete MADS-box transcription factor family in Arabidopsis: new openings to the MADS world Plant Cell 2003 15 1538 1551 10.1105/tpc.011544 12837945 PMC165399

[B64] Du Z Zhou X Ling Y Zhang Z Su Z agriGO: a GO analysis toolkit for the agricultural community Nucleic Acids Res 2010 38 Web Server issue W64 W70 20435677 10.1093/nar/gkq310 PMC2896167

