# Supplementary figures and images for: Moderate drought causes dramatic floral transcriptomic reprogramming to ensure successful reproductive development in Arabidopsis
Source: BMC Plant Biol. 2014 Jun 13;14:164. doi: 10.1186/1471-2229-14-164 (PMC4067085; doi:10.1186/1471-2229-14-164)

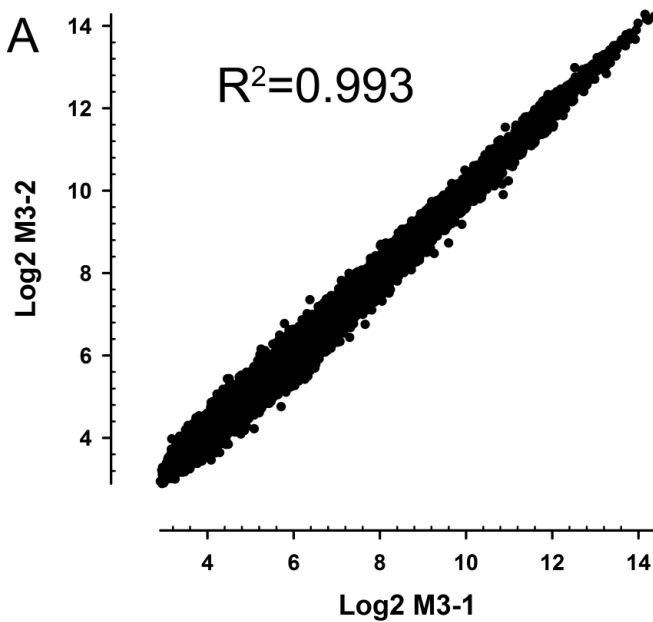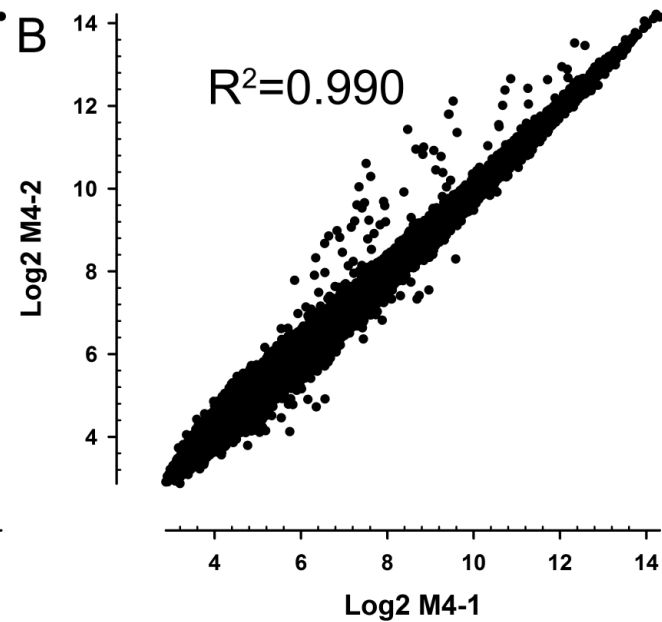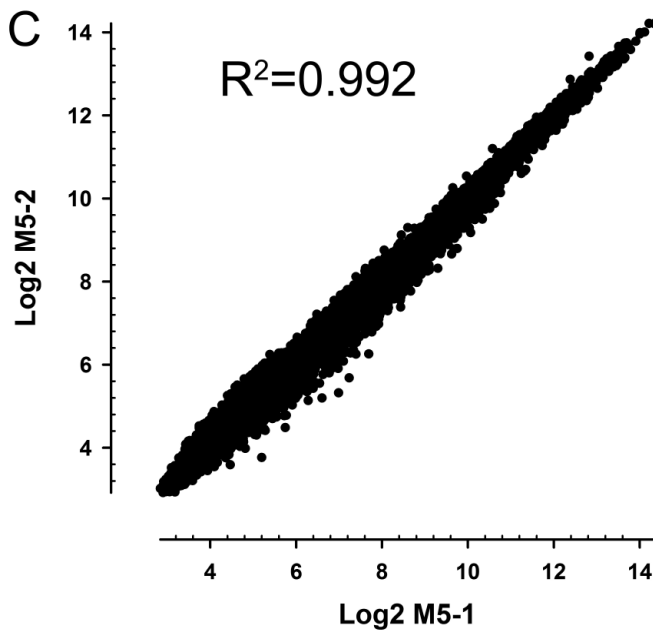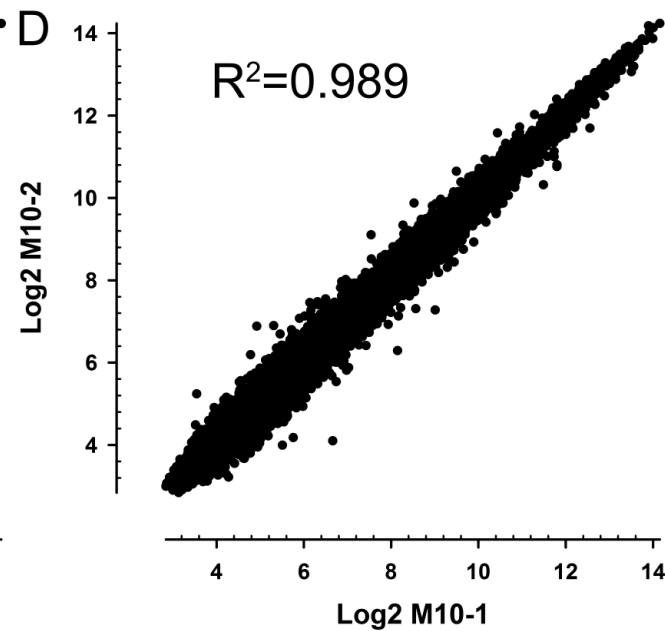

Supplement: Additional file 1 — Microarray data correlation between two biological replicates at each time point. [file 1471-2229-14-164-S1.pdf]

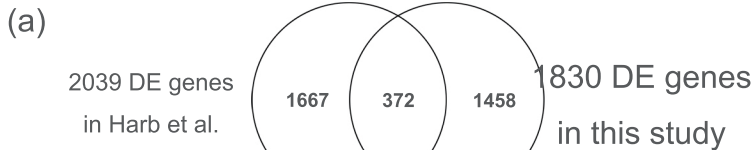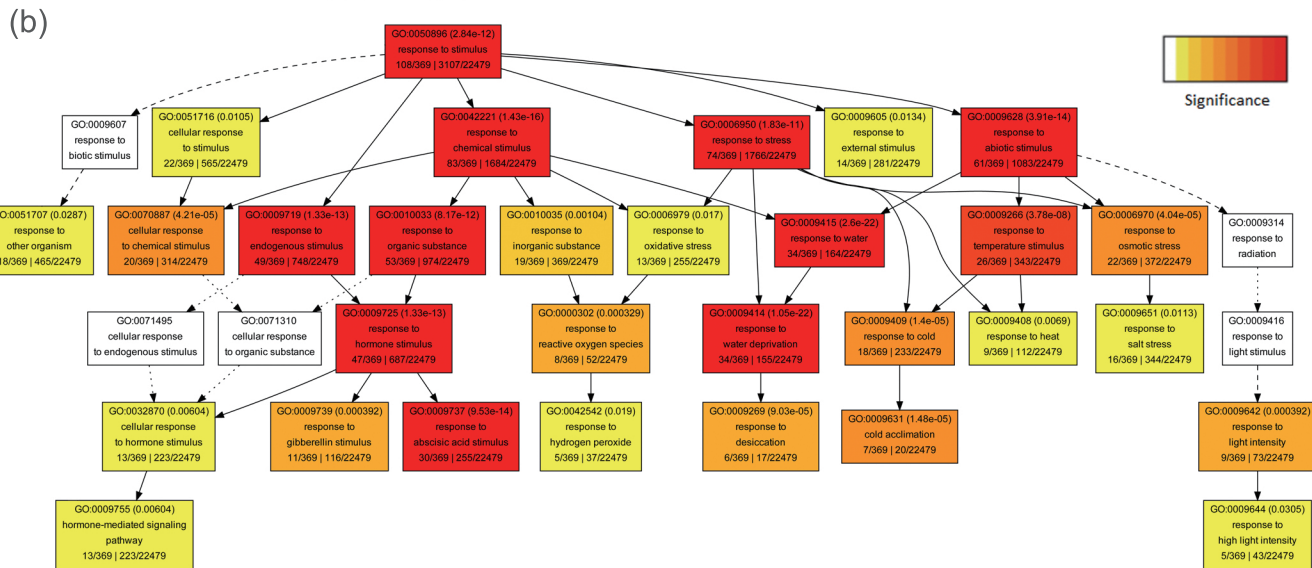

Supplement: Additional file 9 — Venn diagram and GO enrichment analyses of the comparison between our differentially expressed genes with the DE genes from vegetative tissue study by Harb et al. [file 1471-2229-14-164-S9.pdf]
